# MEK1/2-ERK Pathway Alterations as a Therapeutic Target in Sporadic Alzheimer’s Disease: A Study in Senescence-Accelerated OXYS Rats

**DOI:** 10.3390/antiox10071058

**Published:** 2021-06-30

**Authors:** Natalia A. Muraleva, Nataliya G. Kolosova, Natalia A. Stefanova

**Affiliations:** Institute of Cytology and Genetics (ICG), Siberian Branch of Russian Academy of Sciences (SB RAS), 10 Lavrentieva Avenue, 630090 Novosibirsk, Russia; kolosova@bionet.nsc.ru (N.G.K.); stefanovan@mail.ru (N.A.S.)

**Keywords:** Alzheimer’s disease, extracellular regulated kinase (ERK1/2), mitogen-activated protein kinases (MAPK), mitochondria-targeted antioxidant, SkQ1, OXYS rats

## Abstract

Alzheimer’s disease (AD) is a progressive neurodegenerative disorder and the most common cause of dementia worldwide, with no cure. There is growing interest in mitogen-activated protein kinases (MAPKs) as possible pathogenesis-related therapeutic targets in AD. Previously, using senescence-accelerated OXYS rats, which simulate key characteristics of the sporadic AD type, we have shown that prolonged treatment with mitochondria-targeted antioxidant plastoquinonyl-decyltriphenylphosphonium (SkQ1) during active progression of AD-like pathology improves the activity of many signaling pathways (SPs) including the p38 MAPK SP. In this study, we continued to investigate the mechanisms behind anti-AD effects of SkQ1 in OXYS rats and focused on hippocampal extracellular regulated kinases’ (ERK1 and -2) activity alterations. According to high-throughput RNA sequencing results, SkQ1 eliminated differences in the expression of eight out of nine genes involved in the ERK1/2 SP, compared to untreated control (Wistar) rats. Western blotting and immunofluorescent staining revealed that SkQ1 suppressed ERK1/2 activity via reductions in the phosphorylation of kinases ERK1/2, MEK1, and MEK2. SkQ1 decreased hyperphosphorylation of tau protein, which is present in pathological aggregates in AD. Thus, SkQ1 alleviates AD pathology by suppressing MEK1/2-ERK1/2 SP activity in the OXYS rat hippocampus and may be a promising candidate drug for human AD.

## 1. Introduction

Alzheimer’s disease (AD) is a progressive neurodegenerative disorder and the most common cause of dementia worldwide, with no cure. The prevalence of AD is increasing dramatically due to the aging of the world population. At present, there is no effective drug for this complex disease [1], even though the investigation into the mechanisms of AD is very active. There is growing interest in mitogen-activated protein kinases (MAPKs) as potential targets for pathogenesis-directed therapy of AD. Numerous reports have revealed a relationship between the activation of MAPKs and accumulation of pathological aggregates of beta-amyloid (Aβ) and hyperphosphorylated tau protein in neurofibrillary plaques [2,3] as well as neuroinflammation [4], oxidative stress, and other hallmarks of AD. Zhu and coauthors have demonstrated that there are differences in the activation of various MAPKs during AD development in comparison with healthy people [5]. p38 MAPK activity is associated with mild and severe stages of AD, whereas extracellular regulated kinases’ (ERK1/2) activities are altered at all the stages of this disease, including stages with limited pathological signs [5].

MAPK pathways are the key mechanism that transmits extracellular signals, including inflammatory cytokines and reactive oxygen species, from the plasma membrane to the nucleus. Among them, the ERK1/2 pathway is important for the central nervous system [6]. It participates in the regulation of differentiation, maturation, and migration of cells, thereby contributing to the establishment of their phenotype [7]. It is noteworthy that the ERK pathway is involved in the regulation of neurogenesis both during brain formation and in adulthood [8]. The ERK pathway actively participates in mechanisms of synaptogenesis, in the transmission of cellular signals, and in the changes related to neural plasticity, including those linked with learning and memory processes, and configures specific networks for the correct processing of emotional signals [6]. Disturbances of ERK pathway activity are associated with neurological syndromes such as autism [9]. Some of the proteins that give rise to pathological deposits in the brain during AD, including tau protein and Aβ, are cytosolic targets of ERK, which take part in the establishment of pathological hallmarks and in neurodegeneration [10,11]. Faucher and colleagues have shown that Aβ aggregates trigger the ERK1/2 signaling pathway (SP) in the brain at early stages of AD [12]. Other authors have found that ERK1/2 activity is implicated in tau phosphorylation during AD [2,3,13]. Greater amounts of phospho-ERK (p-ERK) have been found in brain extracts from AD patients [14]. Thus, the ERK1/2 SP is strongly implicated in AD development and can become a promising therapeutic target. Currently, there are no effective therapeutic agents targeting the ERK1/2 SP for AD treatment. Researchers are focusing their efforts on the determination of selectivity profiles when designing kinase inhibitors that can cross the blood–brain barrier and on the optimization of their therapeutic index [4]. In the present study, we hypothesized that mitochondria-targeted antioxidant plastoquinonyl-decyltriphenylphosphonium (SkQ1) globally affects the activities of MAPK-related SPs; the ability of SkQ1 to delay the development and suppress the progression of AD-like pathology in a rat model of a sporadic type of the disease has previously been reported by us [15,16,17,18].

This work is continuation of a series of studies on the identification of AD mechanisms and effective targets for devising therapeutic and preventive strategies against AD as a complicated multifactorial disorder. Using nontransgenic senescence-accelerated OXYS rats, which develop neurodegenerative changes that are similar to the signs of the sporadic type (>90% of cases) of AD in humans [19], we have previously reported that treatment with SkQ1 between ages 12 and 18 months—that is, during active progression of AD-like pathology in these animals—alleviates structural neurodegenerative alterations, improves the structural and functional state of mitochondria, prevents the neuronal loss and synaptic damage, enhances a neurotrophic supply, and decreases Aβ_1–42_ peptide levels and tau hyperphosphorylation in the hippocampus, thus resulting in improvements in learning ability and memory [15,16,17]. Via transcriptomic approaches, we then found that the anti-AD properties of SkQ1 are related to improvements in the activities of many intracellular processes and SPs in the prefrontal cortex and hippocampus, including the p38 MAPK SP [20,21], which is launched during the development of AD-like pathology in OXYS rats [22]. In this study, we continued to investigate the mechanisms of the anti-AD effects of SkQ1 in OXYS rats at an advanced stage of AD-like pathology and focused on MEK1/2-ERK pathway alterations in the hippocampus.

## 2. Materials and Methods

### 2.1. Ethics Statement

All experiments were approved by (and conducted in accordance with the guidelines of) the Ethics Committee on animal testing of the Institute of Cytology and Genetics, Novosibirsk, Russia (the decree of the Presidium of the Russian Academy of Sciences No. 12000-496 of 2 April 1980).

### 2.2. Animals and Diet

The effect of SkQ1 dietary supplementation was investigated with senescence-accelerated OXYS rats (with Wistar rats as the control) obtained from the Center for Genetic Resources of Laboratory Animals at the ICG SB RAS (Novosibirsk, Russia), which were kept under standard laboratory conditions. One group (*n* = 8) consumed a diet supplemented with SkQ1 (250 nmol/[kg body weight]) on dried bread slices between ages 12 and 18 months every day. Twelve-month-old OXYS and Wistar rats (*n* = 8) that ingested dried bread without the drug served as controls. SkQ1 was kindly provided by Skulachev Vladimir, from Institute of Mitoengineering of Moscow State University, Moscow, Russia.

### 2.3. Tissue Preparation

Untreated 18-month-old OXYS and Wistar rats and OXYS rats after the dietary supplementation with SkQ1 (*n* = 3) were euthanized by CO_2_ asphyxiation and used for RNA-seq analysis. The right brain hemisphere of the remaining animals in each group (*n* = 5) was excised on ice and fixed under standard conditions (buffered 4% paraformaldehyde for 48 h). Then, the fixed brain was transferred to 30% sucrose for 48 h incubation. The hippocampus from the left hemisphere of the rat brains (*n* = 5 per group) was isolated on ice and put in clean tubes for Western blot analysis. The samples of brain tissue were stored at −70 °C until analysis.

### 2.4. Gene Expression Analysis

RNA isolation, Illumina sequencing, and sequencing data processing were performed as described elsewhere [20]. The genes with an adjusted *p* value of <0.05 were defined as differentially expressed. The list of genes related to ERK1/2 SP was compiled by comparing the gene lists in the Rat Genome Database (RGD; 123 rat genes, https://rgd.mcw.edu/ (accessed on 12 January 2021) and Kyoto Encyclopedia of Genes and Genomes (KEGG; https://www.genome.jp/kegg (accessed on 12 January 2021).

### 2.5. Western Blotting

The hippocampus from OXYS and Wistar rats (*n* = 5 per group) at the age of 18 months after the supplementation with SkQ1 was subjected to Western blotting. Frozen tissue samples of the hippocampus were homogenized in protein radioimmune precipitation buffer (50 mM Tris-HCl pH 7.4, 150 mM NaCl, 1% Triton X-100, 1% sodium deoxycholate, 0.1% sodium dodecyl sulfate, and 1 mM ethylenediaminetetraacetic acid) supplemented with a protease inhibitor cocktail (P8340; Sigma-Aldrich, St. Louis, MO, USA). After incubation for 20 min on ice, the samples were centrifuged at 12,000× *g* for 30 min at 4 °C. The resultant supernatants were collected as a detergent-soluble fraction. The pellets were resuspended in an equal volume of SDS sample buffer, rehomogenized, sonicated, and centrifuged at 14,000× *g* for 10 min at 4 °C. The supernatants were transferred into new tubes as a detergent-insoluble fraction. Total protein was quantified by means of the Bio-Rad Bradford Kit (Bio-Rad Laboratories, Hercules, CA, USA). The protein fractions were transferred onto a nitrocellulose membrane. After blockage with 5% bovine serum albumin (BSA; cat. # SLBJ8588V; Sigma-Aldrich, St. Louis, MO, USA) in phosphate buffered saline with 0.1% Tween 20 for 1 h, the membranes were incubated at 4 °C overnight with one of the following primary antibodies: anti-alpha B crystallin antibody, anti-phospho-S45 CryaB antibody, anti-ERK1/2 antibody, anti-ERK1/2 (phospho T202, T185) antibody, anti-MEK1/2 antibody, anti-MEK1/2 (phospho S218, S222) antibody, anti-tau, anti-phospho-tau (T181), and anti-β-actin and anti-GAPDH antibodies (cat. # ab76467, ab5598, ab184699, ab201015, ab178876, ab194754, ab1801, and ab8245, respectively; Abcam, Waltham, MA, USA; 1:1000 dilution). We incubated the membrane with secondary antibodies (cat. # ab97046 and ab6721; Abcam, Waltham, MA, USA; 1:5000) for 1 h. The intensity of chemiluminescent signals of the bands was quantified using ImageJ software (NIH, Bethesda, MD, USA).

### 2.6. Immunofluorescent Staining

The brain tissue slices (16 μm thick) of OXYS and Wistar rats were placed onto Polysine glass slides (Menzel-Glaser; Thermo Scientific, Braunschweig, Germany) and incubated for 1 h in a blocking solution consisting of 1% BSA (Sigma-Aldrich, St. Louis, MO, USA) in PBS with 0.1% Triton X-100 (PBS-T). The slices were incubated overnight at 4 °C with the same primary antibody (as in Western blotting) diluted 1:250 with the blocking solution. After serial washes in PBS, the slices were probed for 1 h in the dark at room temperature with a secondary antibody (cat. # ab150170, ab175472, and ab150075; Abcam, Waltham, MA, USA; 1:5000) diluted 1:300 in a 1% BSA solution in PBS. Next, the slices were washed with PBS and coverslipped with Fluoro-shield mounting medium containing 4′,6-diamidino-2-phenylindole (DAPI; cat. # ab104139; Abcam, Waltham, MA, USA). Negative controls were processed in an identical manner except that the primary antibody was omitted. The experiment included a negative control. The immunofluorescent signals were visualized by means of an Axioplan 2 fluorescence microscope (Carl Zeiss, Jena, Germany).

### 2.7. Statistical Analysis

The Newman-Keuls post hoc test in Statistica 8.0 software (StatSoft, Tulsa, OK, USA) was applied to significant main effects and interactions in order to evaluate the differences between some sets of means. One-way ANOVA was performed for pairwise group comparisons. The data are presented as mean ± SEM. Differences were regarded as statistically significant at *p* < 0.05.

## 3. Results

### 3.1. Dietary Supplementation with SkQ1 Reverses Up- and Downregulation of the Genes Associated with the ERK1/2 SP in the Hippocampus of OXYS Rats

**Figure 1:**
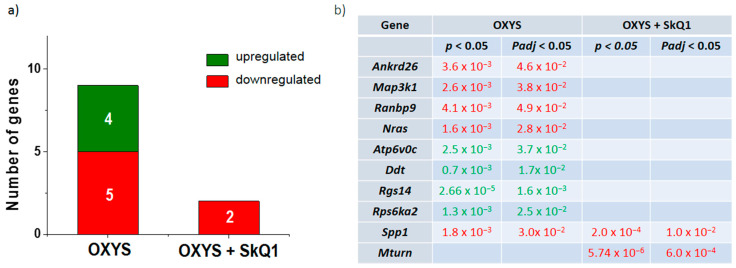
The effects of the dietary supplementation with SkQ1 in OXYS rats on the expression of the genes whose products play a role in the ERK1/2 SP (**a**). Differentially expressed genes of the ERK1/2 SP in untreated (control) OXYS rats and the influence of prolonged treatment with SkQ1 (**b**). Differential expression means a comparison with the parental control strain (Wistar). The data are marked in green if upregulated and red if downregulated of genes. *p*: probability values; *p*_adj_: adjusted *p* values.

Recently, we showed that the hippocampus of 18-month-old OXYS rats features 1159 differentially expressed genes relative to Wistar rats, and that treatment with SkQ1 from age 12 to 18 months decreases their number by twofold [20]. In untreated (control) OXYS rats, the most significant specific feature was the number of differentially expressed genes associated with mitochondrial function, whereas SkQ1 eliminated differences in the expression of 76% of them (93 of 122 genes). Out of ~300 differentially expressed genes whose products participate in SPs, 26 genes encode proteins taking part in MAPK signaling cascades, of which 13 are involved in the p38 MAPK SP (out of 57 known genes of the p38 MAPK SP) [22]. In the present study, the analysis of the ERK1/2 SP identified nine genes (out of 54 known genes of the ERK1/2 SP) whose expression differed in the hippocampus between 18-month-old OXYS and Wistar rats (Figure 1a). The expression of genes *Atp6v0c*, *Ddt*, *Rgs14*, and *Rps6ka2* was higher, while the expression of genes *Ankrd26*, *Map3k1*, *Ranbp9*, *Nras*, and *Spp1* was lower in untreated OXYS rats (Figure 1). Treatment with SkQ1 eliminated the differences in the expression of these genes except for *Spp1*. In addition, SkQ1 decreased the mRNA level of *Mturn* (Figure 1a,b).

### 3.2. SkQ1 Reduces the Amounts of Proteins ERK1 and ERK2 and Their Phosphorylation in the OXYS Hippocampus

**Figure 2:**
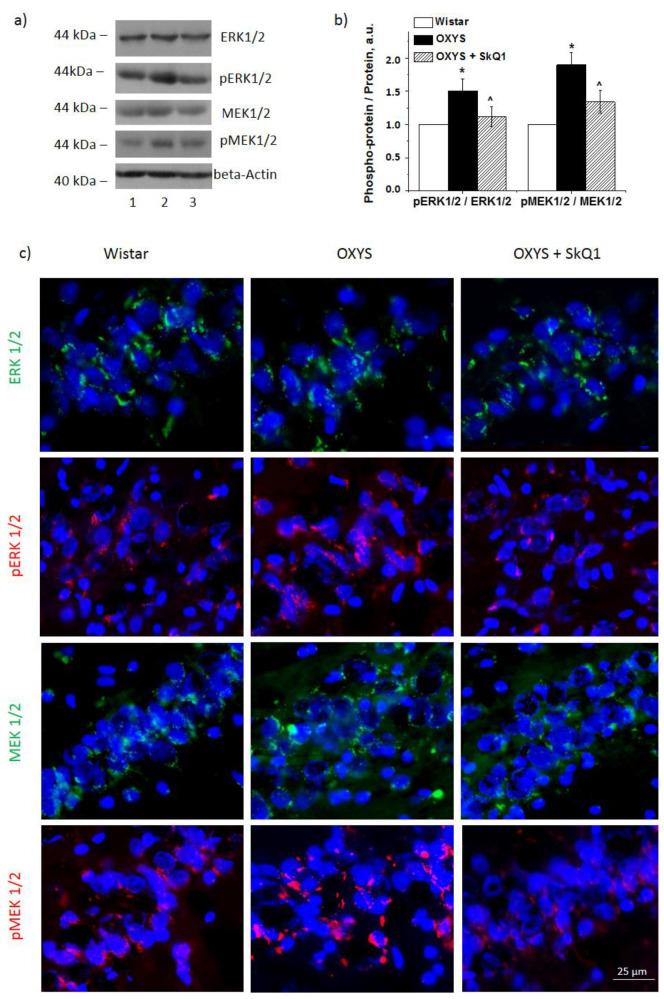
The impact of SkQ1 supplementation in OXYS rats between ages 12 and 18 months on protein levels of ERK1/2 SP components in the hippocampus. (**a**) Representative Western blot images of proteins ERK1/2, p-ERK1/2, MEK1/2, and p-MEK1/2: Wistar rats (1), OXYS rats (2), and OXYS rats after the supplementation with SkQ1 (3). Graphical presentation of the ratios of proteins p-ERK1/2 to ERK1/2 and p-MEK1/2 to MEK1/2 in the hippocampi of untreated Wistar and OXYS rats and in OXYS rats after the supplementation with SkQ1 (**b**). The protein amounts were normalized to beta-actin or GAPDH and then were normalized to the data from Wistar rats. The results are presented as mean ± SEM of five independent experiments. * Statistically significant differences between the strains of the same age; ^ the effect of the supplementation with SkQ1 (*p* < 0.05). Immunostaining of ERK1/2, p-ERK1/2, MEK1/2, and p-MEK1/2 in the hippocampus of untreated Wistar and OXYS rats and OXYS rats taking SkQ1 (**c**). Nuclei were stained with 4′,6-diamidino-2-phenylindole dihydrochloride(DAPI) (blue). Scale bars: 25 µm.

Western blotting revealed that the combined level of proteins ERK1 and ERK2 in the hippocampus was higher in OXYS rats than in Wistar rats (*p* < 0.05; Figure 2a). The combined amount of proteins p-ERK1 and p-ERK2 was significantly higher in OXYS rats than Wistar rats (*p* < 0.05). Figure 2a,b presents the ratio of p-ERK1/2 to total ERK1/2 proteins. These results suggested that the ERK pathway was activated in OXYS rats with age, and the expression of key proteins of the pathway significantly increased.

After the supplementation with SkQ1, ERK1 and ERK2 became less active. SkQ1 had no effect on the combined ERK1 and ERK2 protein level in OXYS rats but reduced their phosphorylation (*p* < 0.05, Figure 2b). One-way ANOVA revealed a decrease in p-ERK1/2 content (*p* < 0.05) in OXYS rats after the supplementation with SkQ1, but it remained higher than that in the control Wistar rats. The ratio of p-ERK1/2 to total ERK1/2 indicated that the supplementation with SkQ1 reduced ERK1/2 phosphorylation (Figure 2b). Similar data were obtained by immunostaining of the brain from untreated Wistar and OXYS rats and OXYS rats treated with SkQ1 (Figure 2c). These findings indicated that the ERK pathway in the hippocampus of OXYS rats was inactivated by the SkQ1 administration.

### 3.3. SkQ1 Reduces MER1/2 and p-MEK1/2 Protein Amounts in the Hippocampus of OXYS Rats

Proteins MEK1 and MEK2 are located upstream of the ERK1/2 SP and regulate the activity of ERK1 and ERK2 by means of their phosphorylation. As expected, we registered increased levels of combined MEK1/2 phosphorylation in 18-month-old OXYS rats compared with Wistar rats (Figure 2a,b). The level of p-MEK1/2 proteins was significantly higher in OXYS rats (*p* < 0.05). Nevertheless, total MEK1/2 content was not different between the two strains.

As displayed in Figure 2a,b, the supplementation with SkQ1 decreased the phosphorylation of MEK1/2 in OXYS rats. Statistical analysis indicated that the decrease in the ratio of p-MEK1/2 to total MEK1/2 was due to a significant decrease in the level of MEK1/2 (*p* < 0.05, Figure 2a). Similar results were obtained after the immunostaining of rat brains (Figure 2c).

### 3.4. SkQ1 Decreases CryaB and p-Ser45-CryaB Protein Amounts in the Hippocampus of OXYS Rats

**Figure 3:**
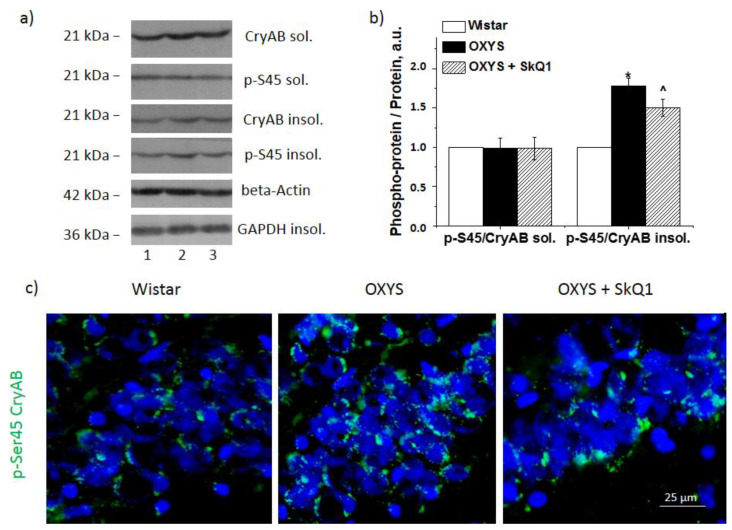
The effect of SkQ1 supplementation in OXYS rats from age 12 to 18 months on the protein levels of CryaB and p-Ser45-CryaB in the hippocampus. Representative Western blots of total and phosphorylated CryaB and p-Ser45-CryaB in the detergent-soluble and detergent-insoluble fractions from the hippocampus of untreated Wistar (1) and OXYS rats (2) and OXYS rats treated with SkQ1 (3) (**a**). Graphical presentation of the ratios of proteins p-Ser45-CryaB to CryaB in the hippocampus of untreated Wistar and OXYS rats and OXYS rats taking SkQ1 after normalization to beta-actin for the detergent-soluble protein fraction and to GAPDH for the detergent-insoluble fraction (**b**). Data are presented as mean ± SEM of five independent experiments. Immunostaining for p-Ser45-CryaB (**c**) in the hippocampus of untreated Wistar and OXYS rats and OXYS rats taking SkQ1. The nuclei were stained with DAPI (blue). Scale bars: 25 µm. ^ Statistically significant differences between the strains of the same age; * the effect of supplementation with SkQ1 (*p* < 0.05). sol.: detergent-soluble protein fraction of hippocampus; insol.: detergent-insoluble protein fraction.

To confirm the inhibitory influence of SkQ1 on the ERK1/2 SP, we estimated the extent of phosphorylation of one of its target proteins—molecular chaperone CryaB—at the Ser45 position. Phosphorylation of CryaB promotes the formation of strong crosslinks with its target proteins and its transfer into a detergent-insoluble fraction. Accordingly, we quantified p-Ser45-CryaB in the detergent-soluble fraction and detergent-insoluble fraction of the protein homogenates. At first, we noted CryaB hyperphosphorylation in the detergent-insoluble fraction of the OXYS rat hippocampus at the age of 18 months (*p* < 0.05; Figure 3a,b). In the detergent-soluble fraction, the CryaB and p-Ser45-CryaB protein amounts did not differ between the strains. The SkQ1 supplementation did not have an impact on CryaB content of the detergent-soluble fraction. The CryaB amount in the detergent-insoluble fraction was lower in SkQ1-treated OXYS rats than in control OXYS rats (*p* < 0.05; Figure 3b). Statistical analysis indicated that SkQ1 significantly reduced the level of p-Ser45-CryaB in the detergent-insoluble protein fractions of the hippocampus from OXYS rats (*p* < 0.05; Figure 3b), but this parameter remained significantly higher than that in untreated Wistar rats (*p* < 0.05). The Western blotting data were confirmed by immunostaining results (Figure 3c).

### 3.5. SkQ1 Prevents Tau Protein Hyperphosphorylation in the Hippocampus of OXYS Rats

**Figure 4:**
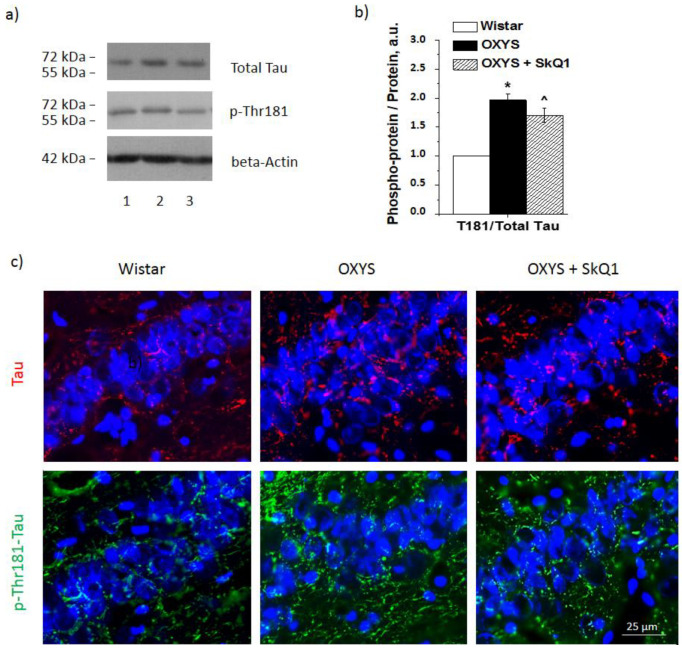
The effects of SkQ1 supplementation in OXYS rats from age 12 to 18 months on protein levels of tau and p-Thr181-tau in the hippocampus. Representative Western blots of total and phosphorylated tau and p-Thr181-tau in the hippocampus of untreated Wistar (1) and OXYS rats (2) and of OXYS rats treated with SkQ1 (3) (**a**). Graphical presentation of the ratios of proteins p-Thr181-tau to tau in the hippocampus of untreated Wistar and OXYS rats and OXYS rats taking SkQ1 after normalization to beta-actin (**b**). Data are presented as mean ± SEM of five independent experiments. Immunostaining for tau and p-Thr181-tau (**c**) in the hippocampus of untreated Wistar and OXYS rats and OXYS rats taking SkQ1. Nuclei were visualized with DAPI (blue). Scale bars: 25 µm. ^ Statistically significant differences between the strains of the same age; * the effect of supplementation with SkQ1 (*p* < 0.05).

According to Western blot findings, OXYS rats after the supplementation with SkQ1 showed significantly lower expression of total tau and p-Thr181-tau (*p* < 0.05 and <0.01, respectively; Figure 4a,b). The Western blotting data were confirmed by immunostaining results (Figure 4c).

## 4. Discussion

As members of the MAPK family, ERK1 and ERK2 perform functions at every stage of the growth and formation of cells, including proliferation, differentiation, migration, senescence, and apoptosis. They are also important components of the response to stress during AD development. Here, we showed for the first time that the progression of AD-like pathology in OXYS rats takes place simultaneously with alterations in the expression of nine genes encoding proteins participating in the ERK1/2 SP, whereas dietary supplementation with SkQ1 normalizes the expression of eight of these genes. In our previous studies, we have demonstrated that after supplementation with SkQ1 in OXYS rats, the expression of 14 genes involved in MAPK signaling cascades changes [20], of which 12 are affiliated with the p38 MAPK SP [21]. In the present study, we found that products of the remaining two genes take part in the ERK1/2 SP. Although we did not notice any changes in mRNA levels of the key genes of this SP, the increased expression of *Rgs14* (regulator of G protein signaling 14) in the hippocampus of untreated OXYS rats is interesting. The product of this gene is a suppressor of synaptic plasticity in CA2 neurons and hippocampus-based learning and memory [23]. There are reports that RGS14 inhibition holds promise for normalizing a cognitive impairment [24].

The activation of the ERK1/2 SP in OXYS rats can be confirmed by overexpression of D-dopachrome tautomerase (*Ddt*). The DDT protein participates in the activation of kinases ERK1 and ERK2 [25], ultimately launching the expression of proinflammatory genes and neuroinflammation. In addition, the observed increased expression of ATPase H^+^-transporting V0 subunit C (*Atp6v0c*) suggests a high demand for autophagy [26]. This is an enzyme transporter that acidifies intracellular compartments, and this alteration is necessary for such processes as receptor-mediated endocytosis and establishment of the proton gradient of synaptic vesicles. The disturbance of *Atp6v0c* expression implies a contribution to the onset and progression of an age-related neurodegenerative disease [27]. Among the analyzed differentially expressed genes with downregulation here, secreted phosphoprotein 1 (*Spp1*, also known as osteopontin) is noteworthy. Recently, it was shown to have a potential role in macrophage-mediated Aβ clearance [28]. Therefore, the reduced expression of *Spp1* can promote and/or exacerbate the accumulation of pathological Aβ aggregates and the progression of neurodegeneration in OXYS rats. It can be assumed that all these alterations contribute to the progression of the AD-like pathology in OXYS rats.

Pathological Aβ accumulation may be a prerequisite for ERK1/2 pathway activation in OXYS rats; this accumulation in OXYS rats starts at age 7 months, i.e., at the stage of AD-like pathology manifestation [19]. Similar activation of ERK in hippocampi of a transgenic animal model of AD has been documented during Aβ accumulation [29]. Moreover, ERK1/2 pathway activation with high levels of kinases p-ERK1 and p-ERK2 has been detected in patients with dementia at various stages of behavioral disturbances [5,14].

Additionally, we quantified kinases upstream of the ERK1/2 SP. As expected, kinases MEK1 and MEK2, which act upstream of this SP, turned out to be activated. Elevated amounts of p-MEK1/2 were found in the hippocampus of 18-month-old OXYS rats in comparison with disease-free age-matched Wistar rats. Kinases MEK1 and -2 are activators of ERK1 and -2 and have very narrow substrate specificity. These observations are consistent with the overactivity of the ERK1/2 SP observed here in OXYS rats at the stage of progression of AD-like pathology signs and are similar to findings about other disease models [29]. It should be noted that MEK-ERK pathway activation may be associated with mutations in BRAF or RAS as its upstream activator [30]. On the other hand, in another study on OXYS rats, we did not find mutations in the genes involved in the ERK1/2 pathway [31].

Cytoplasmic targets of p-ERK ensure proteostasis in the cell. There are 200 substrates of ERK1 and ERK2 that have been identified to date [32,33], including tau protein, whose hyperphosphorylation is regarded as one of the main AD characteristics. Here, we confirmed that the progression of AD-like pathology in OXYS rats is concurrent with increases in both the total level of tau protein and its phosphorylation [16]. These data are consistent with the increased ERK1/2 signaling activity in OXYS rats. Furthermore, as a criterion for assessing ERK1/2 signaling activity, we evaluated changes in ERK1/2-dependent phosphorylation of a small protein chaperone, CryaB. As expected, the increased ERK1/2 signaling upregulated p-Ser45-CryaB in the detergent-insoluble fraction of the 18-month-old OXYS rat hippocampus. The phosphorylation of CryaB enhances its ability to form strong bonds with neurotoxic proteins, including Aβ, and makes CryaB insoluble. Elevated CryaB phosphorylation has also been uncovered by other authors in an AD study, where this phenomenon was associated with pathologically aggregated proteins [34].

Here, we hypothesized that one of the mechanisms behind the beneficial effect of SkQ1 is mediated by ERK1/2 pathway inhibition. Indeed, the OXYS rats on the diet supplemented with SkQ1 manifested a decrease in the p-ERK1/2 level, but not total ERK1/2, in the hippocampus. In addition, we registered decreased phosphorylation of kinases MER1 and -2, which are kinase activators functioning upstream of the ERK1/2 SP. In addition, we noticed decreased phosphorylation of cytosolic targets of this pathway: tau protein and CryaB. A decrease in tau hyperphosphorylation is thought to be a positive prognostic sign during the treatment of AD. Together with a decrease in Aβ accumulation, this change alleviates the toxic load on neurons. Accordingly, the obtained data mean that SkQ1 inhibits the ERK1/2 SP by blocking kinases MER1 and -2.

Activation of ERK1/2 signal transduction is involved in aberrations of the mitochondrial fission/fusion ratio and defects in mitochondrial function. Ganand and coauthors have demonstrated that ERK inactivation normalizes anomalous mitochondrial dynamics in AD [35]. This observation can explain the previously reported strong connection between the neuroprotective property of SkQ1 and an improvement in the structure and functions of mitochondria [17,18] and in the expression of genes related to mitochondrial function [20]. Furthermore, the suppression of ERK1/2 phosphorylation protects neuronal cell lines and primary cultured neurons from direct oxidative stress [31].

ERK1/2 signaling plays an important role in the regulation of synaptic plasticity in AD. Upregulation of total ERK is associated with deficient memory task performance in a transgenic mouse model of AD [23]. Alterations in ERK1/2 signaling induce Aβ-associated behavioral deficits in an animal model of AD [36]. Conversely, ERK1/2 pathway inhibition eliminates memory deficits in a transgenic model of AD [29], in agreement with our finding that SkQ1 has a beneficial impact on the behavior of OXYS rats [15].

Thus, in our study, we demonstrated that active progression of AD-like pathology in OXYS rats is associated with alterations of the ERK1/2 SP. The mitochondria-targeted antioxidant SkQ1, which alleviated AD pathology through MEK1/2-ERK1/2 pathway suppression in the OXYS rat hippocampus, can be regarded as a promising therapeutic agent against human AD.

## Data Availability

Data is contained within the article.
